# Identification of Mucorales by Matrix-Assisted Laser Desorption Ionization Time-of-Flight Mass Spectrometry

**DOI:** 10.3390/jof5030056

**Published:** 2019-07-02

**Authors:** Patrick Schwarz, Houssem Guedouar, Farah Laouiti, Frédéric Grenouillet, Eric Dannaoui

**Affiliations:** 1 Department of Internal Medicine, Respiratory and Critical Care Medicine, University Hospital Marburg, D-35043 Marburg, Germany; 2 Center for Invasive Mycoses and Antifungals, Philipps University Marburg, D-35037 Marburg, Germany; 3 Université Paris Descartes, Faculté de Médecine, AP-HP, Hôpital Européen Georges Pompidou, Unité de Parasitologie-Mycologie, F-75015 Paris, France; 4 Centre Hospitalier Régional Universitaire, Hôpital Jean Minjoz, Sérologies Parasitaires et Fongiques, F-25030 Besançon, France; 5 Working Group Dynamyc, Faculté de Médecine, Hôpital Henri Mondor, F-94010 Créteil, France

**Keywords:** mucorales, mucormycosis, identification, MALDI-TOF mass spectrometry

## Abstract

More than 20 different species of Mucorales can be responsible for human mucormycosis. Accurate identification to the species level is important. The morphological identification of Mucorales is not reliable, and the currently recommended identification standard is the molecular technique of sequencing the internal transcribed spacer regions. Nevertheless, matrix-assisted laser desorption ionization time-of-flight mass spectrometry has been shown to be an accurate alternative for the identification of bacteria, yeasts, and even filamentous fungi. Therefore, 38 Mucorales isolates, belonging to 12 different species or varieties, mainly from international collections, including 10 type or neo-type strains previously identified by molecular methods, were used to evaluate the usefulness of matrix-assisted laser desorption ionization time-of-flight mass spectrometry for the identification of human pathogenic Mucorales to the species level. One to three reference strains for each species were used to create a database of main spectrum profiles, and the remaining isolates were used as test isolates. A minimum of 10 spectra was used to build the main spectrum profile of each database strain. Interspecies discrimination for all the isolates, including species belonging to the same genus, was possible. Twenty isolates belonging to five species were used to test the database accuracy, and were correctly identified to the species level with a log-score >2. In summary, matrix-assisted laser desorption ionization time-of-flight mass spectrometry is a reliable and rapid method for the identification of most of the human pathogenic Mucorales to the species level.

## 1. Introduction

Mucormycosis is a life-threatening infection that is associated with a high mortality rate of about 40% [1]. The diagnosis of the disease remains difficult, and the poor outcome of the infection is related to the virulence of the fungi, and its resistance to most of the systemic antifungal drugs that are currently available [2]. An increase of the incidence of mucormycosis has been observed in the post-voriconazole era [3], and many cases of breakthrough infections in patients treated with voriconazole have been reported [4,5].

Mucormycosis is caused by fungi of the order Mucorales, which is a large group of fungi including very diverse species with worldwide distribution. More than 20 different species belonging to more than 10 genera can be responsible for human disease. It has been clearly shown that the different species have different antifungal susceptibility profiles [6,7,8,9]. Identification to the species level is important for better knowledge of the epidemiology of the disease and for the future optimization of the therapeutic strategies.

The identification of Mucorales to the species level remains difficult and time consuming. Indeed, identification based on classical morphology is not always reliable [10], and often, the expertise of a reference laboratory is needed. The molecular identification of Mucorales is feasible particularly by using the internal transcribed spacer regions of the ribosomal RNA as a target. In Mucorales, the internal transcribed spacer regions have low intraspecies variability and a large interspecies variability, allowing a precise identification [11,12,13]. Therefore, internal transcribed spacer sequencing is the gold standard for identification to the species level of isolates belonging to this group of fungi [14].

Molecular identification requires DNA extraction procedures, PCR, sequencing, and time for analyzing the results. Although most hospitals and diagnostic centers have access to a molecular laboratory, which makes this technique attractive, it is generally not used for the routine identification of all filamentous fungi isolated in clinical microbiology laboratories. In contrast, PCR has been a major advance for the diagnosis of mucormycosis, as it can be directly performed using human samples without prior cultivation of the fungus [14]. Other alternative methods of identification have also been evaluated, such as the identification based on carbohydrate assimilation with commercially available test strips [15]. Nevertheless, the technique remains less specific than internal transcribed spacer sequencing.

Mass spectroscopy using matrix-assisted laser desorption ionization time-of-flight (MALDI-TOF) has been shown to be a good alternative to molecular methods in the setting of a clinical microbiology laboratory. MALDI-TOF mass spectrometry has several important advantages over other identification methods, including simple handling, low costs, speed, and the possibility of high-throughput. MALDI-TOF mass spectrometry has been shown to be very useful for yeast identification [16]. Filamentous fungi such as *Fusarium* spp. as well as *Aspergillus* spp. [17,18,19,20] and to a lesser extent the Mucorales have also been evaluated [18,21,22,23,24,25,26,27].

The aim of the present study was to evaluate the accuracy of MALDI-TOF mass spectrometry for the identification of Mucorales to the species level from pure cultures.

## 2. Materials and Methods

A collection of well-characterized isolates of Mucorales has been used for this study. Isolates were obtained from international fungal collections such as CBS (Westerdijk Fungal Biodiversity Institute, Utrecht, Netherlands) and UMIP (Fungi Culture Collection of Institut Pasteur, Paris) as well as from hospital collections (microbiology laboratory of HEGP, Hôpital Européen Georges-Pompidou, and CHU Besançon, Centre Hospitalier régional Universitaire de Besançon). All the isolates were previously identified to the species level by molecular sequencing of the internal transcribed spacer regions. As far as possible, the type strain for each species was included. A total of 38 isolates from different origins (environmental, animal, and human) and from various geographical areas were included in the study.

### 2.1. Extraction Protocol for MALDI-TOF Mass Spectrometry Analysis

Sample preparation for MALDI-TOF mass spectrometry was performed as previously described with modifications [25]. The isolate was grown on solid medium until good sporulation was obtained. A mixture of spores and mycelium was harvested from the culture with the aid of a sterile cotton swab. The fungal material was transferred to a 1.5-mL microtube containing 300 μL of sterile distilled water, vortexed, and 900 μL 70% ethanol were added. After centrifugation for 10 min at 13,000 rpm, the pellet was resuspended in a mixture containing 50 μL of 70% formic acid and 50 μL of acetonitrile. After centrifugation for another 5 min at 13,000 rpm, 1.5 μL of the supernatant was spotted onto a steel MALDI target plate (Bruker Daltonik GmbH, Germany) and were allowed to dry at room temperature. Each spot was overlaid with 1.2 μL of alpha-cyano-hydroxycinnamic acid in 2.5% of trifluoroacetic acid and 50% of acetonitrile in water (Bruker Daltonik), and air dried at room temperature. Spectra were acquired using a Microflex LT mass spectrometer (Bruker Daltonik). Spectra were recorded in positive linear mode within a mass range from 2.000 to 20.000 Da and were analyzed using the software Flex Analysis (Bruker Daltonik).

### 2.2. Evaluation of Parameters (Medium, Incubation Time)

The influence of different parameters on the quality of spectra was evaluated. The parameters included culture medium and incubation time. Four reference strains (*Lichtheimia corymbifera*, *Lichtheimia ramosa*, *Lichtheimia ornata*, and *Rhizopus arrhizus*) were used for testing these parameters. For medium comparison, three solid media were used: malt extract agar (Fisher Scientific, Illkirch, France), RPMI 1640 agar (Biomérieux, Marcy-l’Étoile, France), and Sabouraud chloramphenicol gentamicin agar (Bio-Rad, Marnes la Coquette, France). For incubation time, two different time points were evaluated: a short incubation time of 2 days allowing profuse mycelium development and moderate sporulation, and a long incubation time of 7 days that allowed profuse sporulation. All the tests were performed at least in two independent experiments with two to four replicates for each strain.

### 2.3. Construction of the Database

The database was constructed by generating main spectrum profiles. For each species, one or two isolates (or three isolates for *L. ramosa*) were used to generate the main spectrum profiles. Strains were cultured on malt extract agar for 7 days at 35 °C (except for *Mucor* species that were cultured at 30 °C). For each strain, five independent cultures were performed, and from each culture, two fungal suspensions were prepared. Extractions were performed as described above, and each sample was spotted in duplicate. Then, 20 independent spectra were generated per strain. After processing and the visual inspection of each spectrum in the software FlexAnalysis, at least 10 spectra were retained to generate the main spectrum profiles by using the software Biotyper 2.0 (Bruker Daltonik) with default parameters. The following strains (*n* = 18, Table 1) were used for the construction of the database: *L. corymbifera* CBS 429.75, *L. corymbifera* CBS 100.31, *L. ramosa* CBS 270.65, *L. ramosa* CBS 269.65, *L. ramosa* CBS 582.65, *L. ornata* CBS 291.66, *Mucor circinelloides* f. *circinelloides* CBS 195.68, *M. circinelloides* CBS 384.95, *Mucor indicus* CBS 226.29, *Rhizomucor miehei* CBS 182.67, *Rhizomucur pusillus* CBS 354.68, *R. pusillus* IP 3.77, *Rhizopus microsporus* var. *chinensis* CBS 631.82, *Rhizopus microsporus* var. *oligosporus* CBS 112589, *R. arrhizus* CBS 112.07, *R. oryzae* IP 4.77, *Syncephalastrum racemosum* CBS 441.59, and *Cunninghamella bertholletiae* CBS 190.84.

### 2.4. Accuracy Test of the Database

For the accuracy test of the database, MALDI-TOF mass spectrometry analysis was performed for a set of isolates (*n* = 20, Table 2), as described above. Each isolate was tested in quadruplicate. Each spectrum was compared to the newly generated database by the MALDI Biotyper software with default parameters. The algorithm generated a log score value ranging from 0 to 3, by comparing peak intensities, positions, and frequencies. Levels of identification were calculated and interpreted according to the manufacturer: a log score value of >2 indicated species identification, a log score value between 1.7–2 indicated genus identification, and a log score value <1.7 indicated a non-reliable identification.

## 3. Results

### 3.1. Evaluation of Parameters (Medium, Incubation Time)

#### 3.1.1. Medium

Spectra were evaluated for three Mucorales species grown on three different culture media after two different incubation times. After both time points, the spectra were similar, but the intensity of the peaks was different on the three media. For both incubation times, higher intensities of peaks were obtained on malt extract agar compared to RPMI 1640 agar and Sabouraud agar (data not shown). After 2 days of incubation, more peaks were obtained on RPMI 1640 agar, whereas at day 7, more peaks were obtained on Sabouraud agar. Nevertheless, the spectra remained similar on the three media, and differences were species-dependent (data not shown). Globally, malt extract agar seemed to be the most reliable medium, and was chosen for the subsequent experiments.

#### 3.1.2. Incubation time

Two incubation times (2 days and 7 days) were further analyzed on malt extract agar. Overall, there were some differences in the presence and intensities of peaks. To ensure good identification of all the species, and as slow-growing species sporulate insufficiently after 2 days of growth, 7 days of incubation were chosen as the endpoint for the database construction.

### 3.2. Construction of the Database

**Figure 1:**
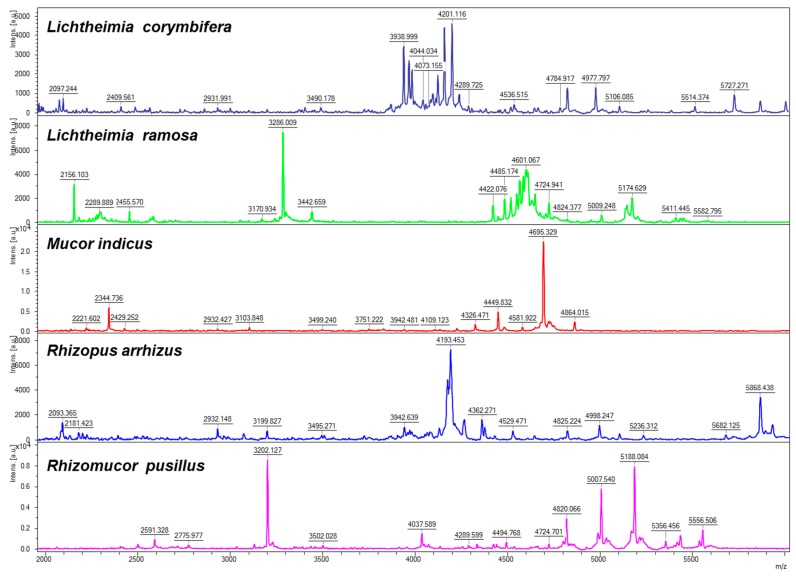
Typical spectra patterns obtained for five different species of Mucorales.

After these preliminary experiments that allowed the determination of the medium and incubation time, the database was set up. For assessing the intergenera, interspecies, and intraspecies variability, all the main spectrum profiles from the database were identified against the database itself. The results of the log-score values are presented in Table 3. A log-score of 3 indicated a perfect match (i.e., when a database strain was compared to itself). A high intergenera variability was observed with a log-score <1.1 for all the strains. The highest similarity between two isolates belonging to different genera was obtained between *M. indicus* and *R. pusillus* (log-score value of 1.01). Similarly, high interspecies variabilities were found for all the species belonging to the *Lichtheimia* (highest log-score of 1.14 between *L. ramosa* and *L. ornata*), *Mucor*, *Rhizomucor*, and *Rhizopus* genus. Examples of spectra of several species are presented in Figure 1, showing the specific patterns for each species. Within a given species, similarity was generally high (log-score up to 2.49 for strains of *L. corymbifera* and 2.82 of *R. pusillus*), but some intraspecies variability was also observed for *L. ramosa*, *M. circinelloides*, *R. microsporus*, and *R. arrhizus*. 

### 3.3. Accuracy Test of the Database

**Figure 2:**
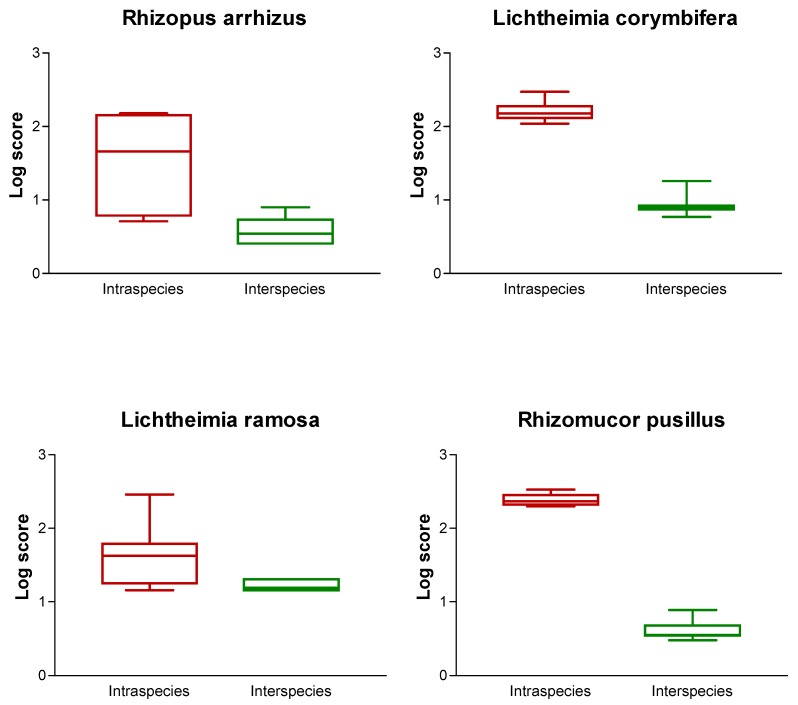
Plot of log-score values obtained for the isolates, belonging to four species, were used for the accuracy test of the database. On each graph, the left panel plots the log-scores values obtained for isolates belonging to the same species (as a marker of intraspecies variability), and the right panel plots the log-scores values obtained for isolates belonging to different species (as a marker of interspecies variability).

Twenty different isolates belonging to the major species (and not used for the construction of the database) were spotted in quadruplicate, and identification was performed using the newly generated database by calculation of the log-score values. Isolates belonged to the most common pathogenic species, namely *R. arrhizus*, *L. corymbifera*, *L. ramosa*, *Mucor indicus*, and *R. pusillus*. The log-score values obtained for all the isolates tested are shown in Table 2. For all the tested isolates, high log-score values of >2 were obtained. For each of the strains, a more detailed analysis was also performed. The 10 best matches were recorded, and log-score values obtained for strains belonging to the same species (measuring the intraspecies variability) and different species (measuring the interspecies variability) were analyzed (Figure 2). For *L. corymbifera* and *R. pusillus*, no overlap of log-score values was seen, indicating that all the tested isolates of these species exhibit a high similarity. In contrast, for *L. ramosa* and *R. arrhizus*, log-score values of <2 were obtained. This indicates that the isolates of these species show a certain degree of interspecies variability. Nevertheless, this did not preclude the correct identification to the species level (Table 2).

## 4. Discussion

MALDI-TOF mass spectrometry is widely used for the identification of bacteria [28] and yeasts [16] to the species level. Major advantages of this identification technique compared to molecular-based identification are its accuracy combined with simplicity, cost effectiveness, and the possibility of high throughput. Nevertheless, the technique also has disadvantages, as the isolates have to be grown as pure cultures, and identification directly from clinical specimens is not possible. Additionally, the identification of filamentous fungi remains challenging, as differences in incubation time, medium selection, and sample preparation may influence the results [29]. Moreover, commercial libraries are incomplete, and often lack a significant number of species and isolates [30]. Therefore, we set up a standardized technique for the sample preparation, and built our own database for the identification of Mucorales to the species level, including reference strains for the most common species responsible for human disease.

Our results demonstrate that a precise discrimination of Mucorales species with a high variability between genera, and also between species, is possible. Intraspecies variability was species-dependent: for some species, a low variability was seen, while for *R. arrhizus* and *L. ramosa*, a certain degree of intraspecies variability was obtained. One limitation of this study is that for some species, only a relatively low number of isolates was included. That might be the reason why for those species, no intraspecies variability was observed. The intraspecies variability of *R. arrhizus* [18,21,22,24,26,27] and *L. ramosa* [25,27] was already reported by others. Our results are in accordance with these findings. Taking these findings into account, it is of major importance to include several isolates of each species into the database to maximize the possibility of reliable identification.

The accuracy of identification of Mucorales to the species level by MALDI-TOF mass spectrometry has already been evaluated by other studies. In one study, a database was set up, including a total of 19 isolates belonging to *Mucor*, *Lichtheimia*, *Rhizopus* spp., and *R. pusillus*. The database was further tested for accuracy with eight Mucorales isolates, including one *R. arrhizus* and four *L. ramosa* strains. No problems in identification were reported; all the log score values were >2 [23]. Another study analyzed the identification of pathogenic *Lichtheimia* species [25]. The database comprised 12 *Lichtheimia* strains belonging to five different species, and seven strains belonging to seven different Mucorales species were also added. The database was tested with 34 additional *Lichtheimia* isolates. All the isolates of *L. corymbifera*, *L. ornata*, *Lichtheimia hyalospora*, and *Lichtheimia sphaerocystis* were correctly identified when taking the highest obtained log-score value into account. When compared to other species, log-score values >2 were obtained, indicating a false identification. The identification of *L. ramosa* strains was even more difficult; log-score values of <1.9 were obtained for two of the 17 tested isolates. Aggravatingly, some isolates exhibited the highest obtained log-score values for a different species, indicating a false identification [25]. Despite the intraspecies variability of *L. ramosa*, in our database, no false identifications were seen for the *Lichtheimia* species. Problems in the identification of *L. ramosa* isolates were also seen in another study evaluating the commercialized Bruker library alone or in combination with an in-house library [27]. The databases were tested with 111 isolates. Despite a significant increase from 49.5% to 81.1% of isolates identified correctly to the species level, when using both libraries compared to the Bruker library alone, seven isolates, including one *L. ornata* and three *L. ramosa* isolates were misidentified. For the other three tested isolates of *L. ramosa*, only an identification to the genus level was possible (log-score value ≥1.7). The intraspecies variability of *L. ramosa* may be related to the existence of at least two different clades within this species, as described previously [31]. In the same study, the identification of *R. arrhizus* isolates was less problematic. Only one of 20 isolates was not identified to the species level [27]. In our study, all three *R. arrhizus* isolates were correctly identified with log-score values >2. Analysis of the spectra of *R. arrhizus* isolates revealed two different spectrum types with different peaks and different peak intensities [22]. Two different types of *R. arrhizus* isolates were also found in another study evaluating the spectra of 14 *R. arrhizus* isolates. Interestingly, the spectra of five *R. arrhizus* isolates were more closely related to 15 *Rhizopus delemar* isolates than to the remaining nine *R. arrhizus* isolates [24]. In a study, evaluating a huge in-house library with 472 filamentous fungal species, including Mucorales, combined with the Burker library, also encountered problems in the identification of *R. arrhizus* isolates. Identification was only possible to the genus level (log-score values >1.7 and <2) [21]. The National Institutes of Health also designed an in-house library containing 152 different mold species, including 27 Mucorales isolates. While no problems were reported when challenged by three *R. arrhizus* isolates, the identification of the same isolates by the Burker database was impossible. Identification of all the other tested Mucorales isolates (n = 9) by the National Institutes of Health database was successful [18]. Problems in the identification of Mucorales to the species level using the Burker database were also reported by others [26]. Globally, different results for the identification of Mucorales to the species level by MALDI-TOF mass spectrometry were obtained, especially for those species showing intraspecies variability. Nevertheless, as long as the database used for identification comprises a sufficient number of reference isolates for each species, identification to the species level seems to be possible.

## 5. Conclusion

We were able to demonstrate that MALDI-TOF mass spectrometry, which is rapid, and cost-effective, is a reliable method for the identification of Mucorales to the species level. It could be useful to implement the database with more clinical and environmental isolates belonging to the different species for even better accuracy.

## Figures and Tables

**Table 1:** List of isolates used for the construction of the database.

Species	Strain Number	Source	ITS Sequence Accession Number
*Rhizopus oryzae*	CBS 112.07^T^	Human, lung	DQ119031
*Rhizopus oryzae*	IP 4.77	Human, brain	DQ119024
*Rhizopus microsporus* var. *oligosporus*	CBS 112589	Environment, tempeh	DQ119011
*Rhizopus microsporus* var. *chinensis*	CBS 631.72^T^	Environment, bread	DQ119009
*Lichtheimia corymbifera*	CBS 429.75^NT^	Environment, soil	FJ719407
*Lichtheimia corymbifera*	CBS 100.31	Animal, aborted cow	FJ19398
*Lichtheimia ramosa*	CBS 269.65	Environment, hay	FJ19405
*Lichtheimia ramosa*	CBS 270.65	Unknown	FJ19406
*Lichtheimia ramosa*	CBS 582.65^NT^	Seed, *Theobroma cacao*	GQ342909
*Lichtheimia ornata*	CBS 291.66^T^	Animal, dung of bird	GQ342891
*Mucor circinelloides* f. *circinelloides*	CBS 195.68^NT^	Environment, air	DQ118991
*Mucor circinelloides*	CBS 384.95^(T)^	Human, skin	DQ119007
*Mucor indicus*	CBS 226.29^T^	Unknown	DQ118994
*Rhizomucor miehei*	CBS 182.67^T^	Environment, plant	DQ118995
*Rhizomucor pusillus*	CBS 354.68^NT^	Environment, cornmeal	DQ119005
*Rhizomucor pusillus*	IP 3.77	Animal, brain of cat	DQ119001
*Syncephalastrum racemosum*	CBS 441.59	Animal, dung	HM999985
*Cunninghamella bertholletiae*	CBS 190.84	Human, heart	HM849701

ITS, internal transcribed spacer; IP, Institut Pasteur; CBS, Westerdijk Fungal Biodiversity Institute; T, type strain; (T), formerly type strain of *Rhizomucor variabilis* var. *regularior*; NT, neo-type strain.

**Table 2:** Identification and corresponding log-score value for 20 Mucorales isolates used for the accuracy test of the database.

Isolate	Collection No.	Identified Species	Score
*Rhizopus arrhizus*	IP 1443.83	*Rhizopus arrhizus*	2.18
*Rhizopus arrhizus*	CBS 120808	*Rhizopus arrhizus*	2.04
*Rhizopus arrhizus*	CBS 120809	*Rhizopus arrhizus*	2.13
*Lichtheimia corymbifera*	IP 1129.75	*Lichtheimia corymbifera*	2.17
*Lichtheimia corymbifera*	IP 1279.81	*Lichtheimia corymbifera*	2.16
*Lichtheimia corymbifera*	IP 1280.81	*Lichtheimia corymbifera*	2.09
*Lichtheimia corymbifera*	CBS 101040	*Lichtheimia corymbifera*	2.21
*Lichtheimia corymbifera*	CBS 120581	*Lichtheimia corymbifera*	2.04
*Lichtheimia corymbifera*	CBS 120580	*Lichtheimia corymbifera*	2.45
*Lichtheimia corymbifera*	BES 335	*Lichtheimia corymbifera*	2.2
*Lichtheimia corymbifera*	PS 1.1	*Lichtheimia corymbifera*	2.07
*Lichtheimia ramosa*	HEGP-3473	*Lichtheimia ramosa*	2.42
*Lichtheimia ramosa*	BES 362	*Lichtheimia ramosa*	2.29
*Lichtheimia ramosa*	BES 228	*Lichtheimia ramosa*	2.46
*Mucor indicus*	CBS 120585	*Mucor indicus*	2.06
*Rhizomucor pusillus*	IP 1127.75	*Rhizomucor pusillus*	2.42
*Rhizomucor pusillus*	IP 1956.90	*Rhizomucor pusillus*	2.38
*Rhizomucor pusillus*	CBS 120587	*Rhizomucor pusillus*	2.42
*Rhizomucor pusillus*	CBS 120586	*Rhizomucor pusillus*	2.53
*Rhizomucor pusillus*	CBS 120588	*Rhizomucor pusillus*	2.31

IP, Institut Pasteur; CBS, Westerdijk Fungal Biodiversity Institute; BES, Centre hospitalier régional universitaire de Besançon; PS; Center for Invasive Mycoses and Antifungals Marburg; HEGP, Hôpital Européen Georges-Pompidou.

**Table 3:** Log-score values obtained when the main spectrum profiles were identified by the database itself. For each isolate, the log-score values for the first 10 best matches are shown.

Isolate Number and Identification	Log-Score Values for Strain																	
	1	2	3	4	5	6	7	8	9	10	11	12	13	14	15	16	17	18
*1- C. bertholletiae* CBS 190.84	3	0.57	0.42	ND	0.43	0.38	ND	0.38	ND	ND	ND	0.52	0.35	ND	ND	ND	0.28	0.58
*2- L. corymbifera* CBS 100.31	0.47	3	2.39	0.85	ND	0.18	0.66	0.6	ND	0.39	ND	ND	ND	0.23	0.35	ND	ND	ND
*3- L. corymbifera* CBS 429.75	0.44	2.41	3	1.14	0.12	0.6	ND	ND	ND	ND	0.48	0.37	0.25	0.26	ND	ND	ND	ND
*4- L. ornata* CBS 291.66	ND	0.8	1.13	3	0.35	0.67	0.67	ND	ND	0.91	ND	ND	ND	0.54	ND	0.4	0.38	ND
*5- L. ramosa* CBS 269.65	0.39	ND	ND	0.58	3	1.78	1.04	0.44	0.21	0.58	ND	ND	ND	0.51	ND	ND	0.37	ND
*6- L. ramosa* CBS 270.35	0.41	ND	0.58	0.6	1.76	3	0.87	ND	ND	ND	0.52	ND	ND	0.4	0.47	ND	0.44	ND
*7- L. ramosa* CBS 582.65	ND	0.42	ND	0.65	1.07	0.98	3	ND	ND	0.27	ND	0.7	0.34	0.38	0.32	ND	ND	ND
*8- M. circinelloides* CBS 195.68	0.58	ND	ND	ND	0.44	0.29	ND	3	0.87	0.98	ND	ND	ND	0.9	0.75	0.28	ND	ND
*9- M. circinelloides* CBS 384.95	0.14	ND	ND	0.1	0.1	ND	ND	0.9	3	0.63	ND	ND	ND	0.76	0.25	ND	0.31	0.13
*10- M. indicus* CBS 226.29	ND	ND	ND	0.95	0.55	0.66	ND	0.98	0.62	3	ND	0.59	0.98	0.53	ND	0.9	ND	ND
*11- R. miehei* CBS 182.67	0.23	0.16	0.04	ND	ND	0.54	ND	ND	ND	0.19	3	0.05	0.27	0.23	ND	ND	ND	0.22
*12- R. pusillus* CBS 354.68	0.48	ND	ND	ND	ND	0.27	0.71	ND	ND	0.57	0.09	3	2.82	0.38	0.64	0.31	ND	ND
*13- R. pusillus* IP 3.77	0.3	ND	0.33	ND	ND	ND	0.35	ND	ND	1.01	0.29	2.82	3	0.33	0.47	0.04	ND	ND
*14- R. microsporus* CBS 631.82	ND	ND	ND	0.61	0.53	0.38	ND	0.94	0.78	0.57	ND	0.38	ND	3	1.54	ND	0.43	ND
*15- R. microsporus* IP 1126.75	ND	ND	ND	ND	ND	0.47	0.31	0.81	0.24	0.42	ND	0.66	0.5	1.54	3	ND	ND	0.3
*16- R. arrhizus* CBS 112.07	ND	ND	ND	0.41	ND	0.66	ND	0.31	ND	0.92	0.05	0.21	ND	ND	ND	3	0.47	0.58
*17- R. arrhizus* IP 4.77	0.33	ND	ND	0.42	0.5	0.45	0.25	ND	0.3	ND	ND	ND	ND	0.45	0.11	0.41	3	ND
*18- S. racemosum* CBS 441.59	0.62	ND	ND	ND	0.16	0.3	ND	ND	0.14	0.3	0.27	ND	ND	ND	0.25	0.81	0.46	3

IP, Institut Pasteur; CBS, Westerdijk Fungal Biodiversity Institute; ND, not determined.
